# Nanoparticle-induced unusual melting and solidification behaviours of metals

**DOI:** 10.1038/ncomms14178

**Published:** 2017-01-18

**Authors:** Chao Ma, Lianyi Chen, Chezheng Cao, Xiaochun Li

**Affiliations:** 1 Department of Mechanical and Aerospace Engineering, University of California at Los Angeles, Los Angeles, California 90095, USA; 2 Department of Engineering Technology and Industrial Distribution, Texas A&M University, College Station, Texas 77843, USA; 3 Department of Mechanical and Aerospace Engineering, Missouri University of Science and Technology, Rolla, Missouri 65409, USA; 4 Department of Materials Science and Engineering, University of California at Los Angeles, Los Angeles, California 90095, USA

## Abstract

Effective control of melting and solidification behaviours of materials is significant for numerous applications. It has been a long-standing challenge to increase the melted zone (MZ) depth while shrinking the heat-affected zone (HAZ) size during local melting and solidification of materials. In this paper, nanoparticle-induced unusual melting and solidification behaviours of metals are reported that effectively solve this long-time dilemma. By introduction of Al_2_O_3_ nanoparticles, the MZ depth of Ni is increased by 68%, while the corresponding HAZ size is decreased by 67% in laser melting at a pulse energy of 0.18 mJ. The addition of SiC nanoparticles shows similar results. The discovery of the unusual melting and solidification of materials that contain nanoparticles will not only have impacts on existing melting and solidification manufacturing processes, such as laser welding and additive manufacturing, but also on other applications such as pharmaceutical processing and energy storage.

## Abstract (web summary)

A material with a deep melted zone (MZ) but small heat-affected zone (HAZ) is ideal for manufacturing, but improving one zone comes at the expense of the other. Here, the authors resolve this contradiction in metals by adding nanoparticles, which change the metals' properties in such a way that both expands MZ and minimizes HAZ.

Melting and solidification are important in the geophysical sciences, food and pharmaceutical processing, energy storage, materials processing and manufacturing^1^. It is thus of scientific and technical interests to control melting and solidification processes. There exists a long-standing dilemma in melting-based processes, such as welding, cladding, surface alloying and additive manufacturing. With the same energy input, a deeper melted zone (MZ) is desired because the material in MZ is well processed, which provides high material performance. In the meantime, a minimal heat-affected zone (HAZ) is ideal because the dissipated heat coarsens the microstructure, which deteriorates the material properties in the HAZ^2^. However, a large MZ generally requires a high energy input and thereby induces a large HAZ, which has remained as a critical challenge in melting-based materials processing technologies. Laser materials processing is used as an example to demonstrate the feasibility of the proposed methodology to solve this dilemma because lasers have been used for manufacturing for decades and have brought remarkable impacts in industries^3^. For example, laser welding has been used for the production of car bodies, railway vehicles and ship structures, providing not only technical advantages, such as deep penetration and full automation, but also economic benefits, such as high efficiency and reliability^4^.

This study is also motivated by the substantial interest in the development of metal matrix nanocomposites (MMNCs), that is, metals containing nanoparticles. There has been an explosive growth in research on MMNCs to unleash their unusual properties for widespread applications^5^,^6^,^7^,^8^,^9^,^10^,^11^,^12^,^13^. It is of interest to use lasers to manufacture and process MMNCs. For example, there is fast-growing research on laser additive manufacturing processes for MMNC products^14^,^15^,^16^,^17^. However, most previous research focused on the mechanical properties changed by nanoparticles while little fundamental understanding has been obtained on the underlining physics of the process.

In the present work, the contradiction between the desired deep MZ and small HAZ is solved by introducing nanoparticles to metals to tune the melting and solidification behaviours. This work is focused on the specific effects of nanoparticles on the heat transfer, melt pool flow and microstructure evolution during laser melting and solidification processes. The nanoparticles are found to increase the MZ while significantly decreasing the HAZ, which tackles a critical challenge in laser manufacturing and other similar processes.

## Results

### Microstructure

**Figure 1:**
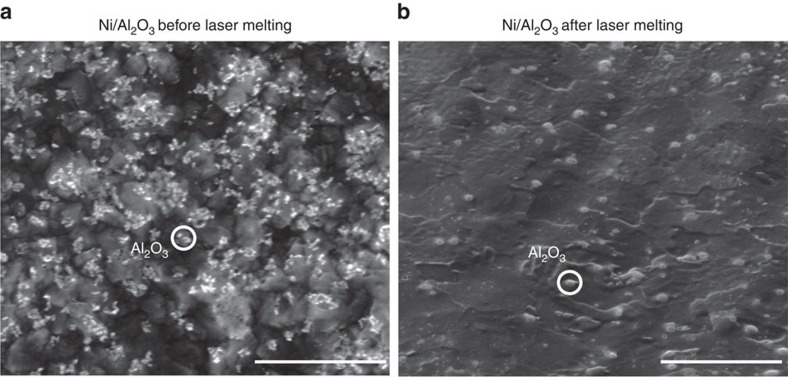
Characterization of Ni/Al_2_O_3_ surface. Scanning electron microscopy (SEM) images before (**a**) and after (**b**) laser melting at a pulse energy of 0.18 mJ. The bright granular phases are the Al_2_O_3_ nanoparticles while the continuous dark phase is the Ni matrix. The nanoparticles are well dispersed and uniformly distributed in the matrix. Scale bars, 1 μm in **a** and 2 μm in **b**.

Pure Ni and Ni/Al_2_O_3_ nanocomposite samples were prepared by electrodeposition and then laser melted using identical parameters. The experimental details are described in the ‘Methods' section. The scanning electron microscopy (SEM) images of the Ni/Al_2_O_3_ surface before and after laser melting at a pulse energy of 0.18 mJ are shown in Fig. 1. The relatively bright phases are the well-dispersed Al_2_O_3_ nanoparticles. The dark phase is the Ni matrix. The fraction of Al_2_O_3_ nanoparticles is 4.4 vol. % and the average diameter is 50 nm. The volume fraction decreased after laser melting. It is possible that some Al_2_O_3_ nanoparticles have been decomposed or ejected from the melt pool.

**Figure 2:**
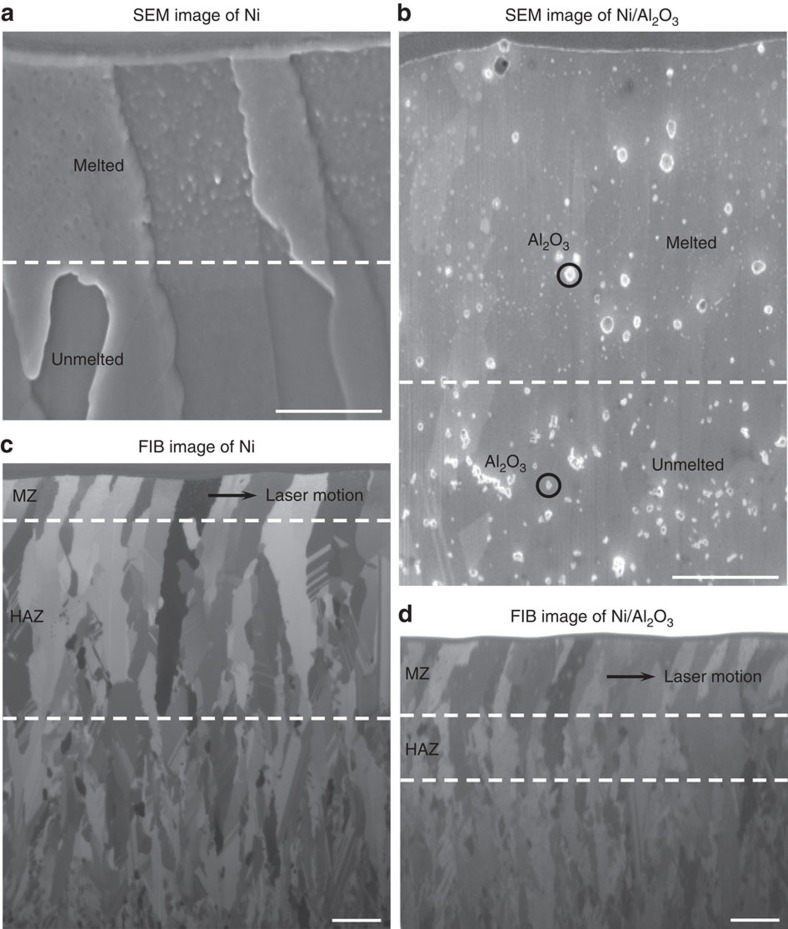
Characterization of Ni and Ni/Al_2_O_3_ cross-sections. Scanning electron microscopy (SEM) images of Ni (**a**) and Ni/Al_2_O_3_ (**b**) that were laser melted at a pulse energy of 0.18 mJ and the corresponding focused ion beam (FIB) micrographs of Ni (**c**) and Ni/Al_2_O_3_ (**d**). The SEM images show the different microstructures between the melted and unmelted zones. The FIB images show the distinct grain structures among the melted zone (MZ), heat-affected zone (HAZ) and the base material. The MZ of Ni/Al_2_O_3_ is deeper than that of Ni, while the HAZ of Ni/Al_2_O_3_ is much smaller than that of Ni. Scale bars, 1 μm in **a**,**b** and 2 μm in **c**,**d**.

For further characterization, the cross-sections of the laser melted samples were cut using focused ion beam (FIB). The SEM and FIB micrographs of the cross-sections of Ni and Ni/Al_2_O_3_ after laser melting are shown in Fig. 2. It should be noted that the thin layer at the very top is a platinum coating deposited before FIB cutting to protect the surface of interest. As shown in Fig. 2a,b, the microstructure of the laser melted zone is different from the unmelted zone for both the materials. In the pure Ni sample, there exist many tiny granular features induced by the laser melting process. In the Ni/Al_2_O_3_ nanocomposite sample, the amount of nanoparticles in the melted zone is less than in the unmelted zone, which agrees with the surface characterization results in Fig. 1. The boundaries are marked by white dashed lines in the images. The melt depth of the Ni/Al_2_O_3_ nanocomposite, 3.2±0.2 μm, is much deeper than that of pure Ni, 1.9±0.2 μm.

Ion beam can induce channelling contrast from different grains because the penetration of the ions depends on the crystallographic orientation of each grain, which makes FIB a powerful tool to characterize grain structures. Figure 2c and d show the FIB micrographs of the Ni and Ni/Al_2_O_3_ cross-sections. Three zones can be identified based on the difference in grain structures from the top to the bottom: (i) large tilted grains, (ii) large vertical grains and (iii) small vertical grains. The first region is the melted zone (MZ), in which the tilted grains due to the asymmetric heating/cooling from the raster scan of the laser. The MZ depths recognized in the FIB images (Fig. 2c,d) are consistent with those from the SEM graphs (Fig. 2a,b). The second region is identified as the heat-affected zone (HAZ), in which the grains are significantly coarsened compared with the original microstructure. The third region is the base material, which remains as the unaffected electrodeposited structures. The boundaries for these three zones are shown as white dashed lines. The HAZ size of Ni/Al_2_O_3_, 2.7±0.2 μm, is remarkably smaller than that of Ni, 8.1±0.3 μm.

**Figure 3:**
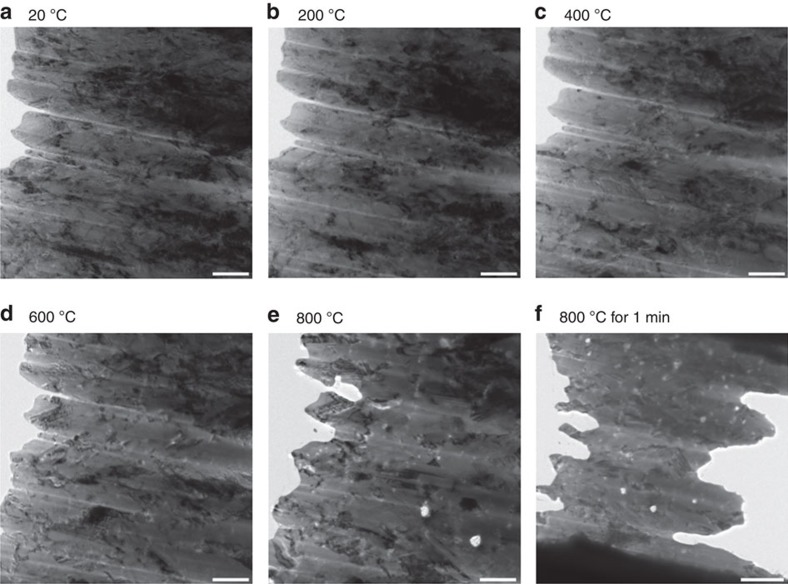
Grain structure evolution of Ni/Al_2_O_3_. Transmission electron microscopy (TEM) images at 20 °C (**a**), 200 °C (**b**), 400 °C (**c**), 600 °C (**d**), 800 °C (**e**) and after 1 min at 800 °C (**f**). The grains of Ni/Al_2_O_3_ appear as columnar and maintain stable until the sample is heated up to 800 °C. The columnar grain boundaries vanish and grow in the through-thickness direction after the temperature increases to 800 °C. Scale bars, 500 nm in **a**–**e** and 1 μm in **f**.

To understand the nanoparticle-enabled stabilization of microstructure in the HAZ, the grain growth behaviour of Ni/Al_2_O_3_ nanocomposite was investigated by transmission electron microscopy (TEM) facilitated with *in situ* heating. Figure 3a shows the microstructure of the as-deposited Ni/Al_2_O_3_. The grain structure of Ni is columnar due to the one-dimensional growth during electrodeposition. The grain structure remained columnar when the sample was heated to 200, 400 and 600 °C, as shown in Fig. 3b–d, respectively, which indicates the grain structure of Ni/Al_2_O_3_ is thermally stable at temperature up to 600 °C. However, when the temperature increased to 800 °C, recrystallization occurred as shown in Fig. 3e. The columnar grain boundaries vanished and the grains transformed to equiaxed grains after holding the sample at 800 °C for about 1 min as seen in Fig. 3f (the image was taken from a lower magnification than other ones to show a larger field). The grains also grew in the through-thickness direction, which resulted in the size reduction of the sample in the view plane. The grain growth temperature of Ni/Al_2_O_3_ is significantly higher than that of Ni, 418 °C (ref. 18), due to the fact that the Al_2_O_3_ nanoparticles effectively inhibit the grain growth. This explains why a large reduction in HAZ was obtained.

### Numerical simulation

**Figure 4:**
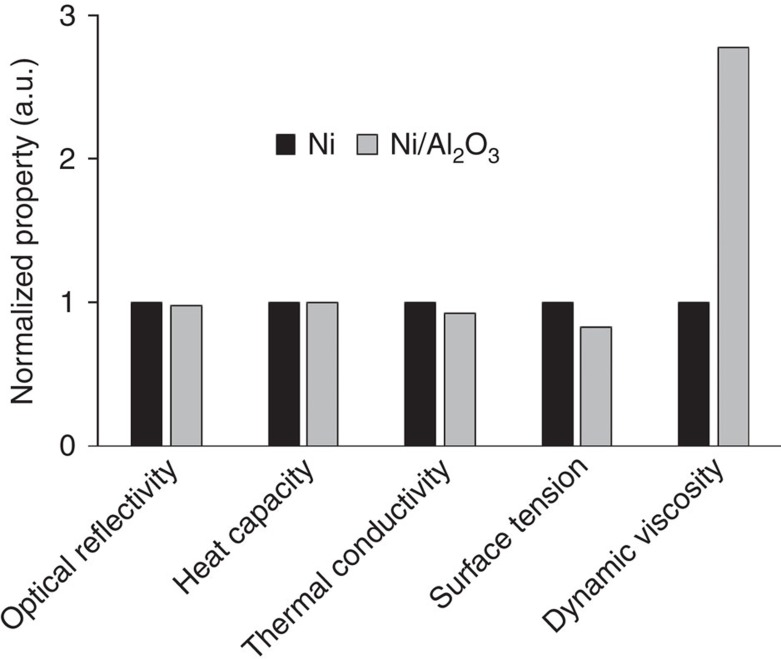
Normalized material properties of Ni and Ni/Al_2_O_3_. The material properties are normalized using Ni as a baseline to show the difference between Ni and Ni/Al_2_O_3_. Optical reflectivity and heat capacity of Ni/Al_2_O_3_ are very close to those of Ni. Thermal conductivity and surface tension of Ni/Al_2_O_3_ are lower than those of Ni while dynamic viscosity of Ni/Al_2_O_3_ is significantly higher than that of Ni.

Recent studies have shown that thermophysical properties (for example, thermal conductivity, surface tension and viscosity) of a material can be altered by addition of nanoparticles^19^,^20^,^21^,^22^. Since a melting process is determined by the thermophysical properties, it is highly possible that the nanoparticle-modified thermophysical properties induced the deeper melting pool. To better understand why nanoparticles induced a smaller HAZ but a deeper MZ, the effects of nanoparticles on optical and thermophysical properties were measured with the experimental methods developed recently^23^,^24^. All the properties that are essential to study laser interaction with nanocomposites were determined, including optical reflectivity, heat capacity, thermal conductivity, surface tension and dynamic viscosity. To show the difference in the properties between Ni and Ni/Al_2_O_3_, the measured material properties are normalized using those of Ni as baselines, which are plotted in Fig. 4. The optical reflectivity and heat capacity are not affected much by the nanoparticles while the thermal conductivity and surface tension are reduced and the dynamic viscosity is significantly increased. Some details of the measurement results are presented in Supplementary Note 1 and Supplementary Fig. 1.

With all these critical material properties available, numerical simulations were performed to understand the effects of nanoparticles on the heat transfer and fluid flow processes and thus the mechanisms for the metallurgical changes. A coupled heat transfer and fluid flow model was developed and validated for laser melting of metals in previous study^25^. It is a two-dimensional axisymmetric transient model based on finite element method. The model is used in the present work to predict the effects of nanoparticles on the heat transfer and fluid flow (that is, Marangoni flow) by using the nanoparticle-modified thermophysical properties. Basically, the nanoparticles change the thermophysical properties and consequently the heat transfer and fluid flow. The input parameters for the numerical model are the actual process parameters and the measured optical and thermophysical properties. The values of these parameters and properties are summarized in Supplementary Table 1. The model predicts the transient temperature field as a function of time and location, based on which the evolution of the MZ and the HAZ can be derived.

**Figure 5:**
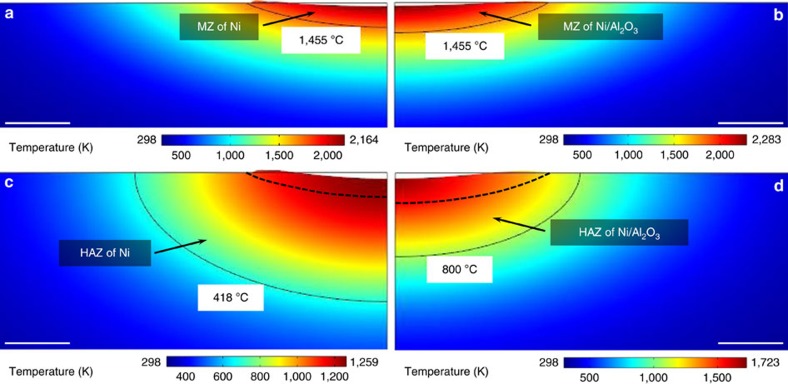
Simulated melted zone (MZ) and heat-affected zone (HAZ) of Ni and Ni/Al_2_O_3_. The predicted MZs of Ni (**a**) and Ni/Al_2_O_3_ that were laser melted at a pulse energy of 0.18 mJ (**b**) and the corresponding HAZs of Ni (**c**) and Ni/Al_2_O_3_ (**d**). The predicted MZ of Ni/Al_2_O_3_ is deeper than that of Ni, whereas the predicted HAZ of Ni/Al_2_O_3_ is much smaller than that of Ni, which agrees well with the experimental results in Fig. 2. Scale bars, 5 μm in **a**–**d**.

The predicted results for the Ni and Ni/Al_2_O_3_ samples are shown in Fig. 5. The implication from the model coincides with the experimental results: the MZ is increased while the HAZ is decreased by the nanoparticles. The numerical model successfully captures the effects of nanoparticles on the heat transfer, melt pool flow and microstructure development using the effective optical and thermophysical properties.

**Figure 6:**
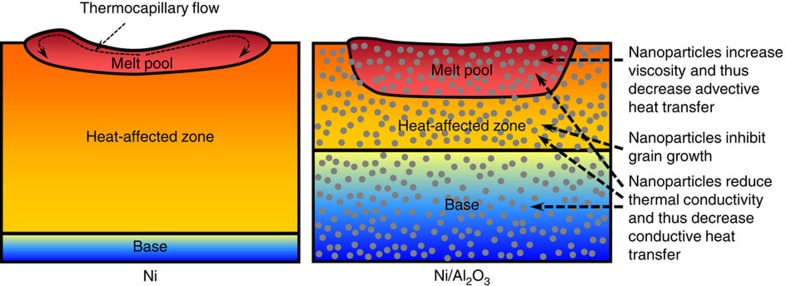
Mechanisms for metallurgical modification by nanoparticles. The nanoparticles decrease the advective and conductive heat transfer to the bulk material and increase the heat accumulation within the melt pool, thereby increasing the melted zone (MZ) depth. The nanoparticles also prevent the grain growth and thus decrease the heat-affected zone (HAZ) size.

The numerical simulation is insightful to understand the mechanisms responsible for the evolution of the distinct microstructures between Ni and Ni/Al_2_O_3_. The model shows that the maximum surface temperature of Ni/Al_2_O_3_ is higher than that of Ni, indicating that the nanoparticles deepen the MZ by increasing the heat accumulation within the surface layer. The mitigation of the heat dissipation is further attributed to two mechanisms: (i) the nanoparticles reduce the thermal conductivity and thus decrease the conductive heat transfer to the base material and (ii) the nanoparticles significantly increase the viscosity and thereby suppress the thermocapillary flow and diminish the advective heat transfer within the melt pool. The mechanisms for the increased MZ and decreased HAZ by the nanoparticles are schematically summarized in Fig. 6.

### Effects of nanoparticle material and laser energy

**Figure 7:**
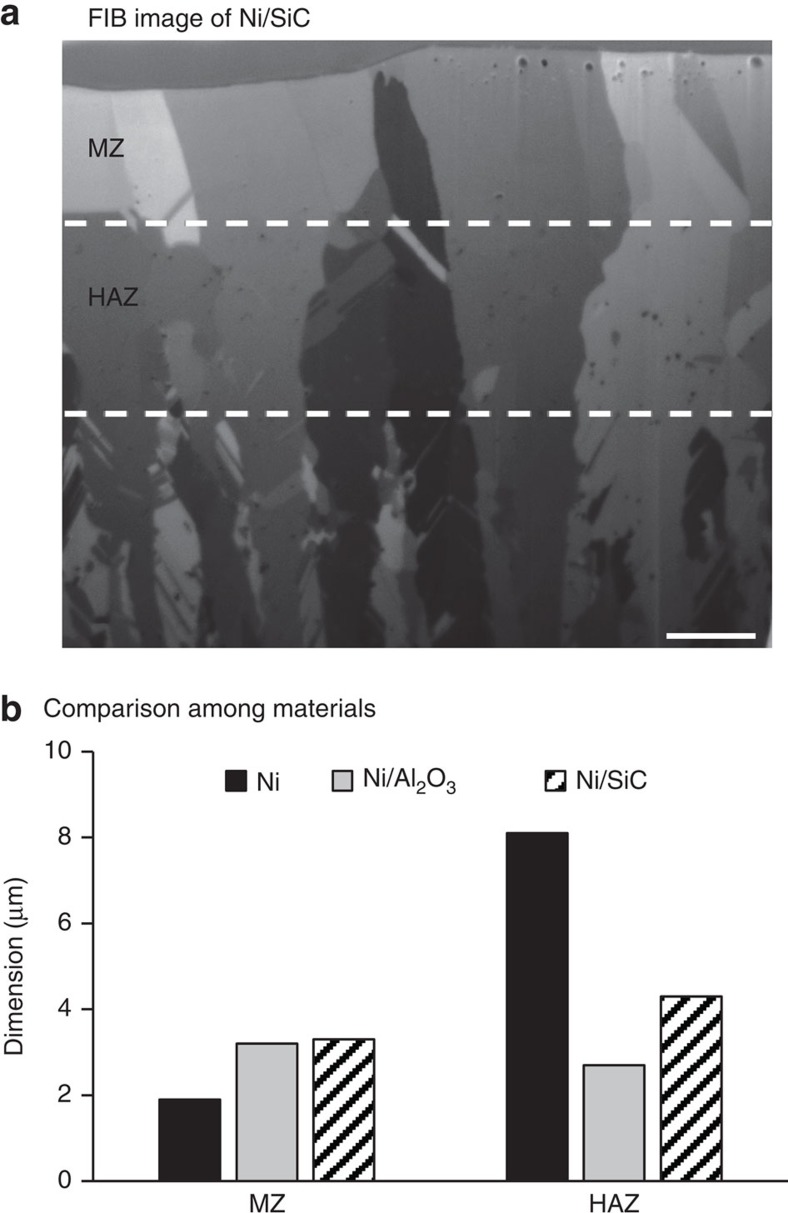
Melted zone (MZ) and heat-affected zone (HAZ) of Ni/SiC. Focused ion beam (FIB) micrographs of Ni/SiC that was laser melted at a pulse energy of 0.18 mJ (**a**) and comparison with those of Ni and Ni/Al_2_O_3_ (**b**). The SiC nanoparticles have the same effects as the Al_2_O_3_ ones: the presence of the nanoparticles increases the MZ depth while decreasing the HAZ size. Scale bar, 2 μm in **a**.

To further validate that it was the nanoparticles that drove the metallurgical changes, the laser melting experiment was also conducted on Ni/SiC nanocomposite. The fraction of the SiC nanoparticles was 3.6 vol. % and the average diameter was also 50 nm. The experimental conditions were the same as those used for Ni and Ni/Al_2_O_3_. As shown in Fig. 7, the presence of SiC nanoparticles has the same effect as that of Al_2_O_3_ nanoparticles, that is, the nanoparticles increased the MZ and reduced the HAZ. Measurements of thermophysical properties were also performed for Ni/SiC. Thermal conductivity was reduced with the addition of SiC nanoparticles while dynamic viscosity was significantly increased. The specific measurement results are presented in Supplementary Note 1 and Supplementary Fig. 1. The effects of SiC nanoparticles on the thermophysical properties and the microstructure are consistent with those of Al_2_O_3_ nanoparticles.

**Figure 8:**
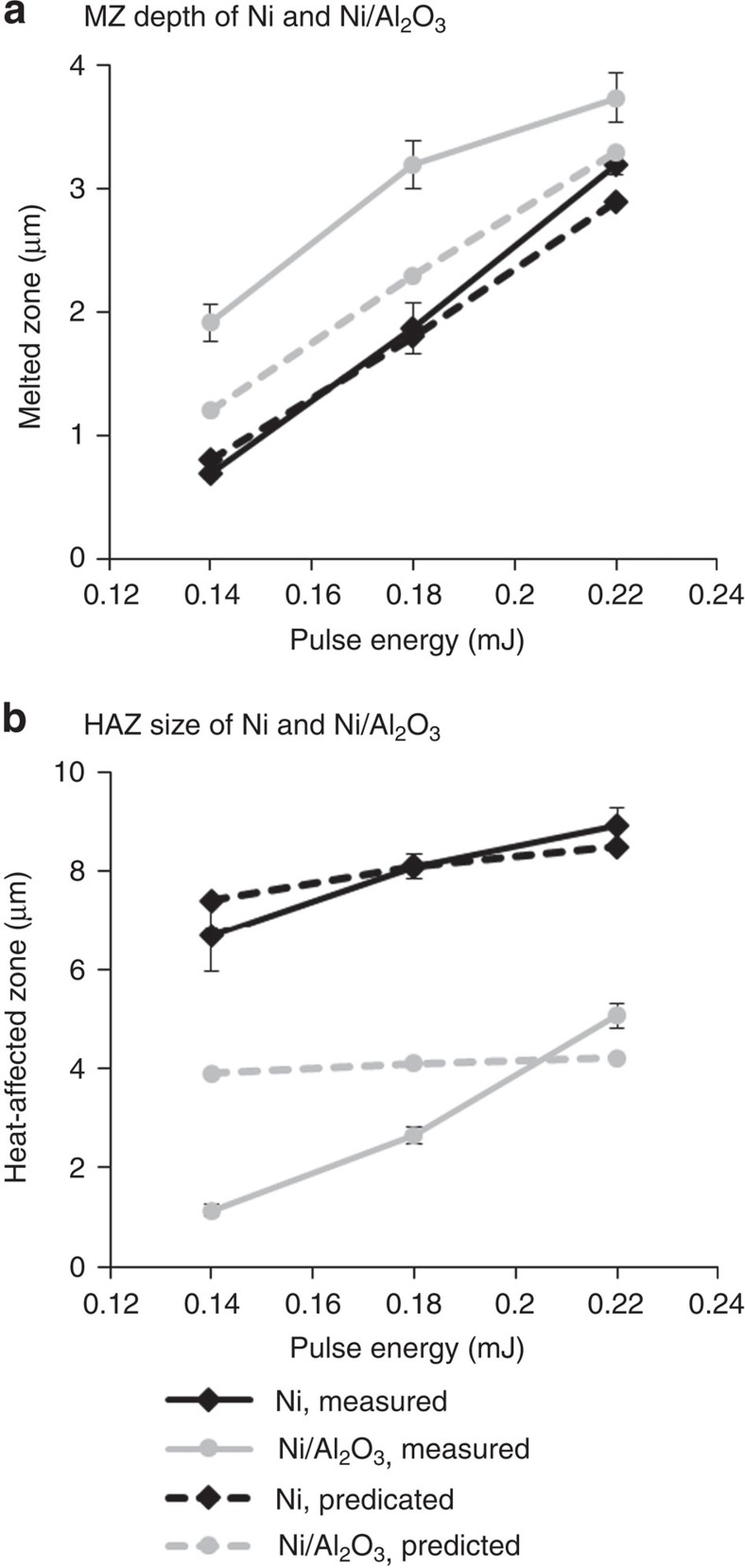
Melted zone (MZ) depth and heat-affected zone (HAZ) size of Ni and Ni/Al_2_O_3_. Measured and predicted MZ depth (**a**) and HAZ size (**b**) of Ni and Ni/Al_2_O_3_ at different pulse energies. As the pulse energy increases, the MZ and HAZ become larger for both materials. The MZ of Ni/Al_2_O_3_ is larger than that of Ni, whereas the HAZ of Ni/Al_2_O_3_ is much smaller than that of Ni at each energy level. The numerical model successfully predicts the effects (trends) introduced by nanoparticles on MZ and HAZ. The error bars in the measured MZ depths and HAZ sizes are the standard deviations calculated over five measurements.

In addition, the effects of laser pulse energy on the MZ depth and HAZ size were studied. Both Ni and Ni/Al_2_O_3_ were laser melted at different energies (0.14–0.22 mJ), followed by the SEM and FIB characterization. The micrographs at different energies are shown in Supplementary Figs 2 and 3. In addition, the numerical simulations were performed at these energy levels. The measured and predicted MZ depth and HAZ size of Ni and Ni/Al_2_O_3_ are plotted as functions of pulse energy in Fig. 8. The MZ and HAZ are enlarged for both materials as the pulse energy increases. The Al_2_O_3_ nanoparticles have the same effect at each energy level, that is, reducing the HAZ while increasing the MZ.

The predicted trends of the MZ and the HAZ for Ni and Ni/Al_2_O_3_ agree well with the experimental results, although there are some differences in the exact values between the predictions and the measurements, especially for Ni/Al_2_O_3_. Laser interactions with metals with the presence of nanoparticles involve rather complex physical phenomena. The numerical model does not take all the effects into account. For example, the laser melting process can change the fraction and the morphology of the nanoparticles and the interfacial bonding between the matrix and the nanoparticles, and thus the thermophysical properties^26^. Also, the thermophysical properties change with temperature, but the temperature-dependent properties of the nanocomposite are not available for the simulation. In addition, the model uses a set temperature to predict the HAZ without considering the kinetics of the grain growth, which is probably the reason why the predicted increasing effects of pulse energy on HAZ are not as substantial as the experimental measurements. In reality, as the laser energy increases, not only does the region above the grain growth temperature increase, but also the duration for grain growth increases, which is not considered in the model. Despite these limitations of the numerical model, it provides insight into understanding the mechanisms of metallurgical changes introduced by the nanoparticles, which is the main objective of the simulation.

In conclusion, the microstructural and numerical studies showed that the nanoparticles reduced the heat dissipation in the laser melting process for a deeper MZ and meanwhile restricted the grain coarsening for a much smaller HAZ, which resolved a long-standing dilemma in the field of manufacturing. Although the present work demonstrates the feasibility to improve manufacturability of materials by nanoparticles, it will be interesting to perform a systematic study in the future on the effects of the material, size, morphology and fraction of nanoparticles on the thermophysical properties of nanocomposites and thus their manufacturability.

It will be of significance if this phenomenon can be extended to other laser processing technologies, such as laser welding and laser additive manufacturing, where the deterioration of the microstructures and material properties of HAZ is a serious problem that limits the performance of the products. Although laser additive manufacturing is based on the melting of powder materials layer by layer and appears different from laser melting, both technologies share the same process physics, including heat transfer, fluid flow and microstructure evolution. Therefore, the proposed approach is applicable to laser additive manufacturing, that is, nanoparticles can be used to tune the melting and solidification processes and thereby improve the manufacturability of the materials. The approach will add another degree of freedom to enhance the capability and extend the application space of a manufacturing process.

## Methods

### Sample preparation

Ni/Al_2_O_3_ and Ni/SiC nanocomposites were prepared by electro-codeposition of Ni and the corresponding nanoparticles in a standard nickel Watts bath containing 10 g l^−1^ of spherical nanoparticles with an average diameter of 50 nm. The solution was mechanically mixed and ultrasonically processed for 2 h before electro-codeposition to achieve a uniform distribution and effective dispersion of the nanoparticles. Electro-codeposition was carried out for 2 h with the continuation of mechanical stirring and sonication to maintain the nanoparticle dispersion and distribution. The temperature was controlled at 43 °C and the current density was set at 1 A dm^−2^. Pure Ni was also prepared under the same conditions for the comparison study.

### Laser melting

The experimental setup of laser melting is schematically shown in Supplementary Fig. 4. A 1,070 nm, 200 W fibre laser (SPI Lasers SP-200C-A-S6-A-C) was directed into a scan head (ScanLab HurryScan III 14 mm). The scan head was furnished with an f-theta objective with a focal length of 163 mm, which resulted in a beam diameter of ∼49 μm at the focal plane. The laser was pulsed at 100 kHz with a pulse duration of 1.5 μs. The laser pulse energy was varied from 0.14 to 0.22 mJ. A raster scan with a track spacing of 4 μm and a scan speed of 0.4 m s^−1^ was performed to cover a square of 1 mm × 1 mm. Argon flow was applied during laser melting to minimize the oxidation. Pure Ni, Ni/Al_2_O_3_, and Ni/SiC nanocomposites were laser melted to investigate the effects of nanoparticles on melting and solidification behaviours.

### Sample characterization

Characterization of microstructures of Ni/Al_2_O_3_ and Ni/SiC was first carried out using scanning electron microscopy (SEM) facilitated with energy dispersive X-ray spectroscopy (EDS/EDX). EDS analysis was used to quantify the fraction of nanoparticles incorporated into the nanocomposites. The volume fraction of nanoparticles was estimated based on the atom fraction of the major element in the nanoparticles (Al or Si) and the base metal (Ni).

To characterize the melted zone (MZ) and the heat-affected zone (HAZ) of Ni, Ni/Al_2_O_3_, and Ni/SiC, the samples were sectioned using focused ion beam (FIB) milling, followed by SEM and FIB imaging. The MZ and HAZ were identified based on microstructural changes. The MZ was determined with two methods: (i) it was identified using SEM by the reduction of the nanoparticles in Ni/Al_2_O_3_ and Ni/SiC nanocomposites and the introduction of tiny granular features in the pure Ni by laser melting and (ii) it was identified by the FIB characterization as the re-solidified titled grains due to uneven heating and cooling. Both the methods agreed with each other. The HAZ was identified by the FIB imaging as the region with considerably coarsened grains compared with the original microstructure. The SEM or FIB image was first divided into five even strips perpendicular to the surface, after which the MZ or HAZ was determined on each strip. The mean and the standard deviation over the five measurements were then calculated and reported.

*In situ* heating transmission electron microscopy (TEM) characterization of Ni/Al_2_O_3_ was facilitated by a micro-heater (DENSsolutions, Delft, Netherlands). The Ni/Al_2_O_3_ sample for TEM was prepared by FIB milling.

### Data availability

All relevant data are available from the authors.

## Additional information

**How to cite this article:** Ma, C. *et al*. Nanoparticle-induced unusual melting and solidification behaviours of metals. *Nat. Commun.* **8,** 14178 doi: 10.1038/ncomms14178 (2017).

**Publisher's note**: Springer Nature remains neutral with regard to jurisdictional claims in published maps and institutional affiliations.

## Supplementary Material

Supplementary Information Supplementary Figures, Supplementary Table, Supplementary Note and Supplementary References.
