# Supplementary material for: Nanoparticle-induced unusual melting and solidification behaviours of metals
Source: Nat Commun. 2017 Jan 18;8:14178. doi: 10.1038/ncomms14178 (PMC5253640; doi:10.1038/ncomms14178)
Supplement: Supplementary Information — Supplementary Figures, Supplementary Table, Supplementary Note and Supplementary References. [file ncomms14178-s1.pdf]

Supplementary Figures

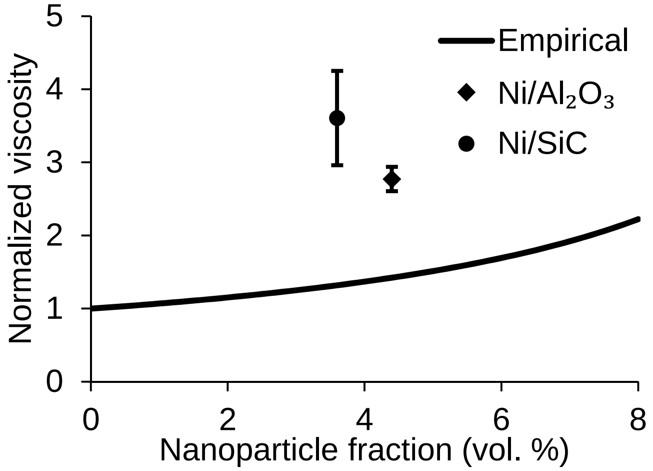

Supplementary Figure 1 | Measured and empirically predicted viscosities. The empirical correlation[1] underestimates the viscosities of Ni/Al_2O_3 and Ni/SiC possibly because the model does not consider the materials of the nanoparticles and the potential bonding between the nanoparticles and the matrix. The error bars in the measured viscosities are the standard deviations cacualted over three measurements.

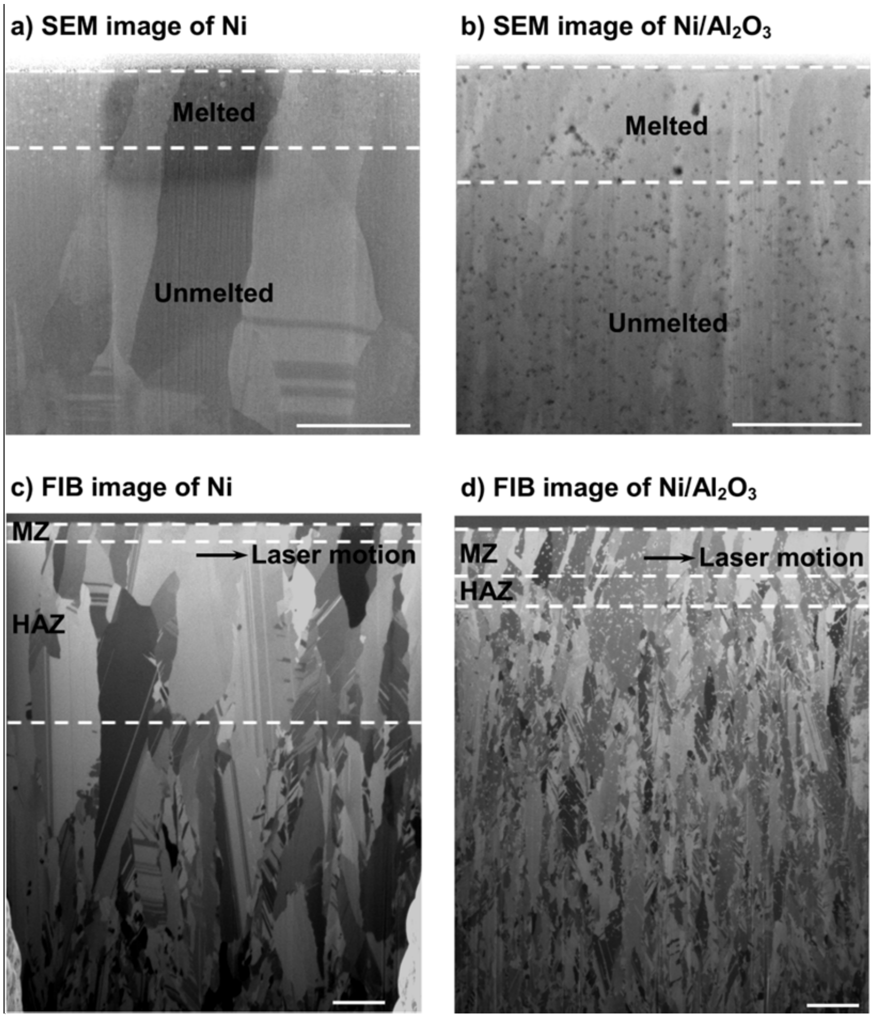

Supplementary Figure 2 | Characterization of Ni and Ni/Al_2O_3 cross sections. Scanning electron microscopy **(**SEM) images of Ni (**a**) and Ni/Al_2O_3 (**b**) that were laser melted at a pulse energy of 0.14 mJ and the corresponding focused ion beam (FIB) micrographs of Ni (**c**) and Ni/Al_2O_3 (**d**). The SEM images show the different microstructures between the melted and unmelted zones. The FIB images show the distinct grain structures among the melted zone (MZ), heat affected zone (HAZ) and the base material. The MZ of Ni/Al_2O_3 is deeper than that of Ni while the HAZ of Ni/Al_2O_3 is much smaller than that of Ni. Scale bars, 1 μm in (**a**) and 2 μm in (**b – d**).

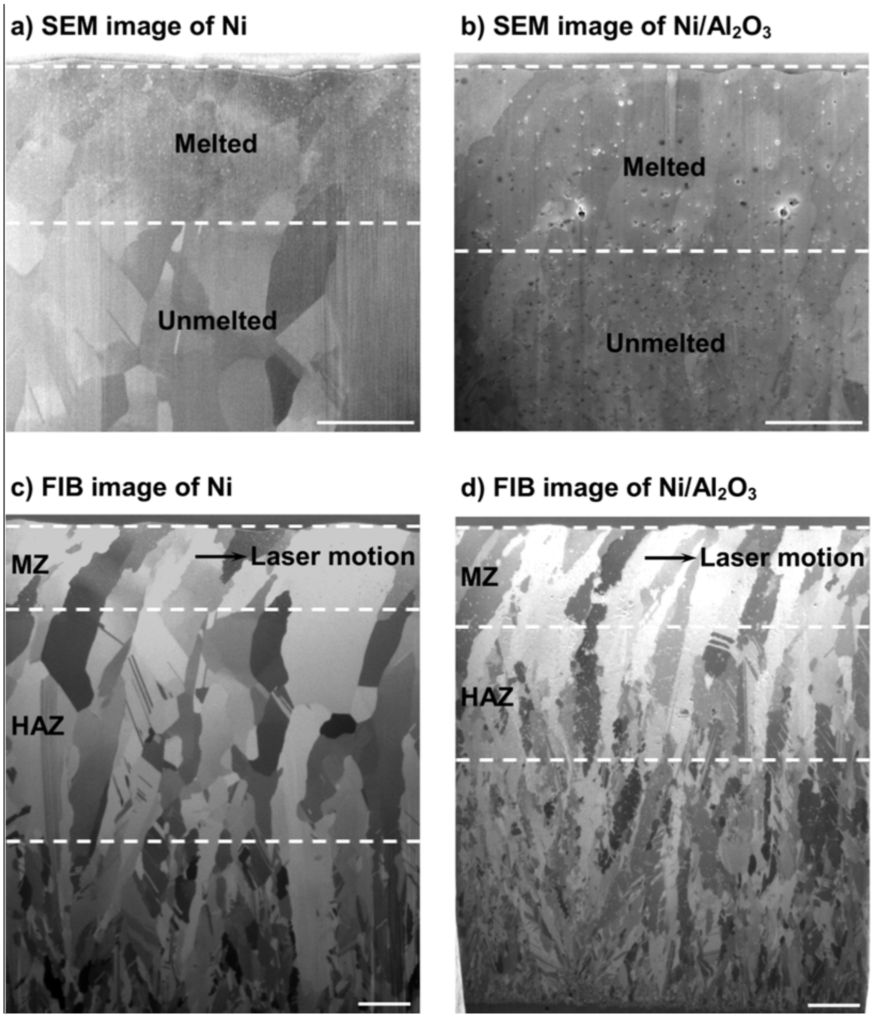

Supplementary Figure 3 | Characterization of Ni and Ni/Al_2O_3 cross sections. Scanning electron microscopy **(**SEM) images of Ni (**a**) and Ni/Al_2O_3 (**b**) that were laser melted at a pulse energy of 0.22 mJ and the corresponding focused ion beam (FIB) micrographs of Ni (**c**) and Ni/Al_2O_3 (**d**). The SEM images show the different microstructures between the melted and unmelted zones. The FIB images show the distinct grain structures among the melted zone (MZ), heat affected zone (HAZ) and the base material. The MZ of Ni/Al_2O_3 is deeper than that of Ni while the HAZ of Ni/Al_2O_3 is much smaller than that of Ni. Scale bars, 2 μm in (**a – d**).

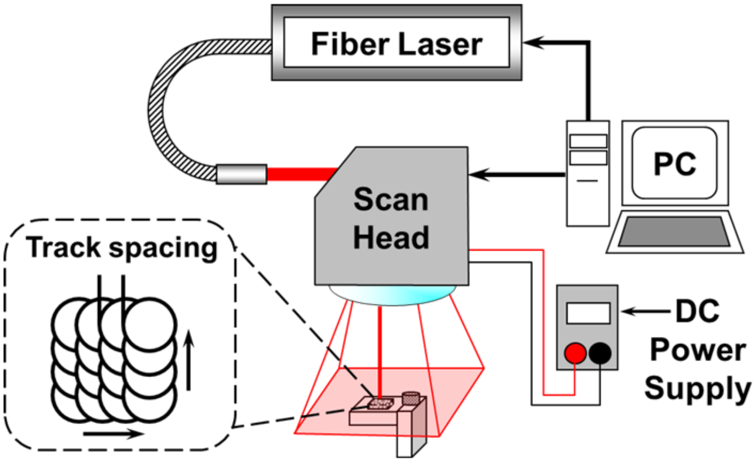

Supplementary Figure 4 | Experimental setup for laser melting. A fiber laser is directed into a scan head for raster scanning. Both units are controlled a computer to allow for synchronization of the scanning motion and the laser irradiation.

Supplementary Table

Supplementary Table 1 | Input parameters for the numerical modelling.

Input Parameter	Ni	Ni/Al_2O_3
Reflectivity (%)	67.4	65.8
Specific heat (J kg^{-1} K^{-1})	424	424
Thermal conductivity (W m^{-1} K^{-1})	91.7	84.1
Dynamic viscosity (mPa s)	4.8	13.3
Laser pulse duration (µs)	1.5	1.5
Beam diameter (µm)	49	49
Pulse energy (mJ)	0.14, 0.18, 0.22	0.14, 0.18, 0.22

Supplementary Note

Supplementary Note 1 | Thermophysical Property Measurements

The measurements of optical and thermophysical properties of the Ni/Al_2O_3 and Ni/SiC nanocomposites used in this work were recently published elsewhere[2,3]. Firstly, using a power meter, the reflectivity of Ni/Al_2O_3 was measured to be 65.8 % while that of Ni was 67.4%, indicating that the Al_2O_3 nanoparticles did not change the reflectivity substantially. Differential scanning calorimetry was used to determine the heat capacity. The specific heat capacities of Ni/Al_2O_3 and Ni/SiC at room temperature were 424 ± 13 J kg^{-1} K^{-1} and 423 ± 14 J kg^{-1} K^{-1}, close to that of Ni, 424 ± 8 J kg^{-1} K^{-1}. An experimental setup was developed to measure thermal conductivity based on the laser flash method. The thermal conductivities of Ni/Al_2O_3 and Ni/SiC at room temperature were 84.1 ± 3.4 W m^{-1} K^{-1} and 87.3 ± 3.4 W m^{-1} K^{-1}, slightly less than that of pure Ni, 91.7 ± 2.8 W m^{-1} K^{-1}. In order to determine the surface tension and viscosity of MMNCs, an innovative measurement system was developed based on the accessible laser system, inspired by the so-called oscillating drop method. The surface tensions of Ni/Al_2O_3 and Ni/SiC at ~1500 °C were 1.39 ± 0.03 N m^{-1} and 1.57 ± 0.06 N m^{-1}, slightly lower than that of Ni, 1.68 ± 0.04 N m^{-1}. The viscosities of Ni/Al_2O_3 and Ni/SiC at ~1500 °C were 13.3 ± 0.8 mPa s and 17.3 ± 3.1 mPa s, significantly higher than that of Ni, 4.8 ± 0.3 mPa s.

The measured viscosities of nanocomposites were compared with an existing empirical correlation obtained from experiments on water/alcohol-based nanofluids containing ceramic/metal nanoparticles[1]. The correlation considers the size and the fraction of the nanoparticles but not the material. As shown in Supplementary Figure 1, the correlation underestimates the viscosities of the Ni/Al_2O_3 and Ni/SiC possibly because the model does not take into account that interfacial bonding may be formed between the liquid metal and the

ceramic nanoparticles at high temperature.

Supplementary References

1 Corcione, M. Empirical Correlating Equations for Predicting the Effective Thermal Conductivity and Dynamic Viscosity of Nanofluids. *Energy Conversion and Management*, 52, 789–793, (2011).

2 Ma, C., Zhao, J., Cao, C., Lin, T., & Li, X. Fundamental Study on Laser Interactions with Nanoparticles-Reinforced Metals — Part I: Effect of Nanoparticles on Optical Reflectivity, Specific Heat, and Thermal Conductivity", *Journal of Manufacturing Science and Engineering* **138**, 121001, (2016).

3 Ma, C., Zhao, J., Cao, C., Lin, T., & Li, X. Fundamental Study on Laser Interactions with Nanoparticles-Reinforced Metals — Part II: Effect of Nanoparticles on Surface Tension, Viscosity and Laser Melting. *Journal of Manufacturing Science and Engineering* **138**, 121002, (2016).
